# Putting Large Language Models into Environmental Context

**DOI:** 10.1021/acs.est.6c06045

**Published:** 2026-10-13

**Authors:** Jinyue Jiang, Jun-Jie Zhu, Zhiyong Jason Ren

**Affiliations:** † School of Sustainable Engineering and the Built Environment, 7864 Arizona State University, Tempe, Arizona 85287, United States; ‡ Department of Civil and Environmental Engineering and Andlinger Center for Energy and the Environment, 6740 Princeton University, Princeton, New Jersey 08544, United States

**Keywords:** Environmental domain model, Large language model, Agentic system, Multimodal learning, Knowledge grounding, Trustworthy AI

## Abstract

The emergence of large language models (LLMs) has fundamentally transformed information interaction, yet their application in environmental science and engineering (ESE) is hindered by multimodal data formats, dynamic context requirements, and hallucination risks. To transition from general-purpose models to domain expert agentic systems, this Perspective introduces a three-pillar framework for ESE-LLMs: domain perception, knowledge grounding, and action capability. Implementing these capabilities introduces potential pitfalls such as geographic representation bias, catastrophic forgetting, and source authenticity vulnerabilities, which require strategic model blending and careful data curation to overcome. Furthermore, rigorous evaluation must extend beyond standard knowledge benchmarks to systematically assess factuality, fairness, consistency, and safety using both human and automated metrics. Finally, we present a decision-guided deployment workflow that helps researchers in ESE fields integrate LLMs into their workflow. Ultimately, achieving trustworthy environmental intelligence relies on continuous evaluation, robust governance, and sustained interdisciplinary collaboration to safely deploy agentic ESE-LLMs for high-stakes environmental challenges.

## Graphical abstract

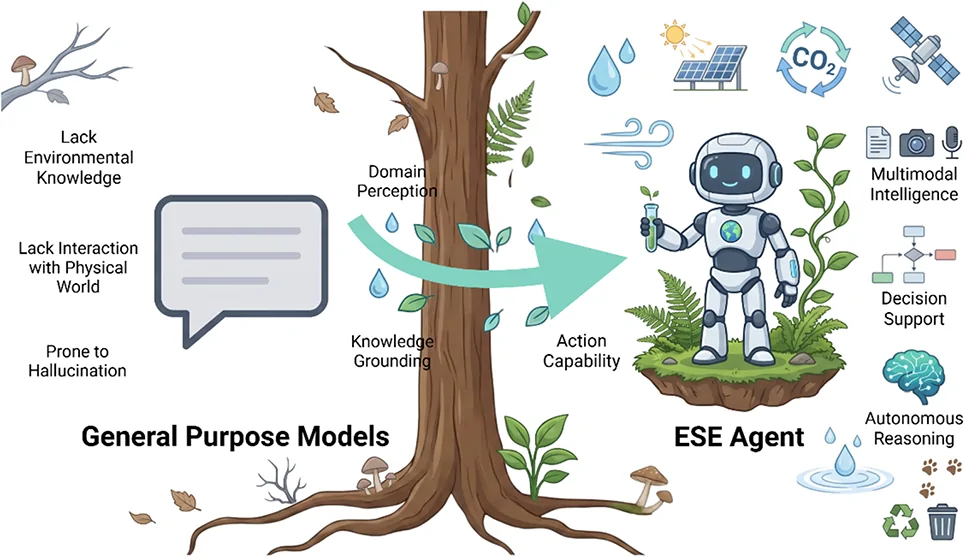

## Introduction

The emergence of large language models (LLMs) has fundamentally transformed our interactions with information. These models, characterized by massive parameter counts and training regimes based on internet-scale corpora, have demonstrated “horizontal competence”—a generalized ability to understand and generate natural language across a vast array of topics.^1–3^ Driven by this trend, significant efforts have been made to integrate LLMs into specialized domains such as medicine and law. Similarly, researchers in environmental science and engineering (ESE) have made efforts to train, fine-tune, or prompt-engineer LLMs for application in the environmental field.^4,5^ However, this form of “vertical intelligence”—the ability to execute complex tasks within a specialized domain—remains nascent compared to the rapid evolution of general-purpose models. Existing research often uses LLMs to accelerate a specific pipeline or repetitive task, while lacking in-depth, domain-specific integrations. In this perspective, we systematically evaluate the need, the methodologies, and the pitfalls associated with developing specialized environmental domain LLMs (ESE-LLMs), identifying pathways toward environmental domain AI agents.

Several distinctive characteristics of ESE explain why general-purpose LLMs often struggle to perform sophisticated tasks in this field. First, environmental problems and data are inherently multimodal and spatiotemporal. For example, environmental professionals may integrate heterogeneous information, including sensor readings, laboratory measurements, biomonitoring data, remote sensing imagery, time-series observations, and complex software interfaces, which differ substantially from the natural language on which most LLMs are trained. While traditional machine learning (ML) and deep learning (DL) models can extract patterns from individual modalities, they lack the semantic reasoning needed to synthesize disparate data streams, such as linking sensor anomalies with regulatory knowledge, into actionable decisions. Consequently, general-purpose LLMs require multimodal adaptation for ESE applications. Furthermore, environmental tasks demand dynamic data and context awareness. In contrast, general-purpose LLMs suffer from a “knowledge cutoff” problem, where parametric memory is limited to information available before training. Therefore, a model estimating national average grid carbon intensity may rely on outdated data, compromising current analyses. ESE is also highly interdisciplinary, requiring the integration of knowledge from fields such as physics, chemistry, microbiology, structural biology, materials science, among others. Moreover, unlike open-ended conversational domains, environmental outputs are anchored in physical reality such as mass balance and thermodynamic laws, and quantities carry units and measurement uncertainty. The field’s ground truth therefore often resides in mechanistic models and monitoring records rather than in text. Finally, hallucination in generative AI remains a critical concern, as models are rarely trained to acknowledge their lack of knowledge. Fabricated information or terminology pose significant risks in the ESE field, where decisions directly impact public welfare. Therefore, ESE-LLMs and their derived AI frameworks must prioritize trustworthy reasoning and hallucination mitigation.

## From General-Purpose Chat-Bot to Domain Expert AI Systems

The booming LLM field has catalyzed many different techniques spanning the entire cycle of LLMs—from initial architectural design to inference-stage techniques such as prompt engineering. We envisioned a three-pillar framework that supports an evolution from generic LLM to ESE-LLM: domain perception, knowledge grounding, and action capability (Figure 1). Rather than introducing new techniques, the framework maps established LLM methods to the unique constraints of the ESE. Specifically, nontextual environmental data require multimodal perception; dynamic regulatory and monitoring data require grounding in authoritative, versioned sources; and physically consistent calculations require action capabilities that delegate computation to code and mechanistic models.

**Figure 1:**
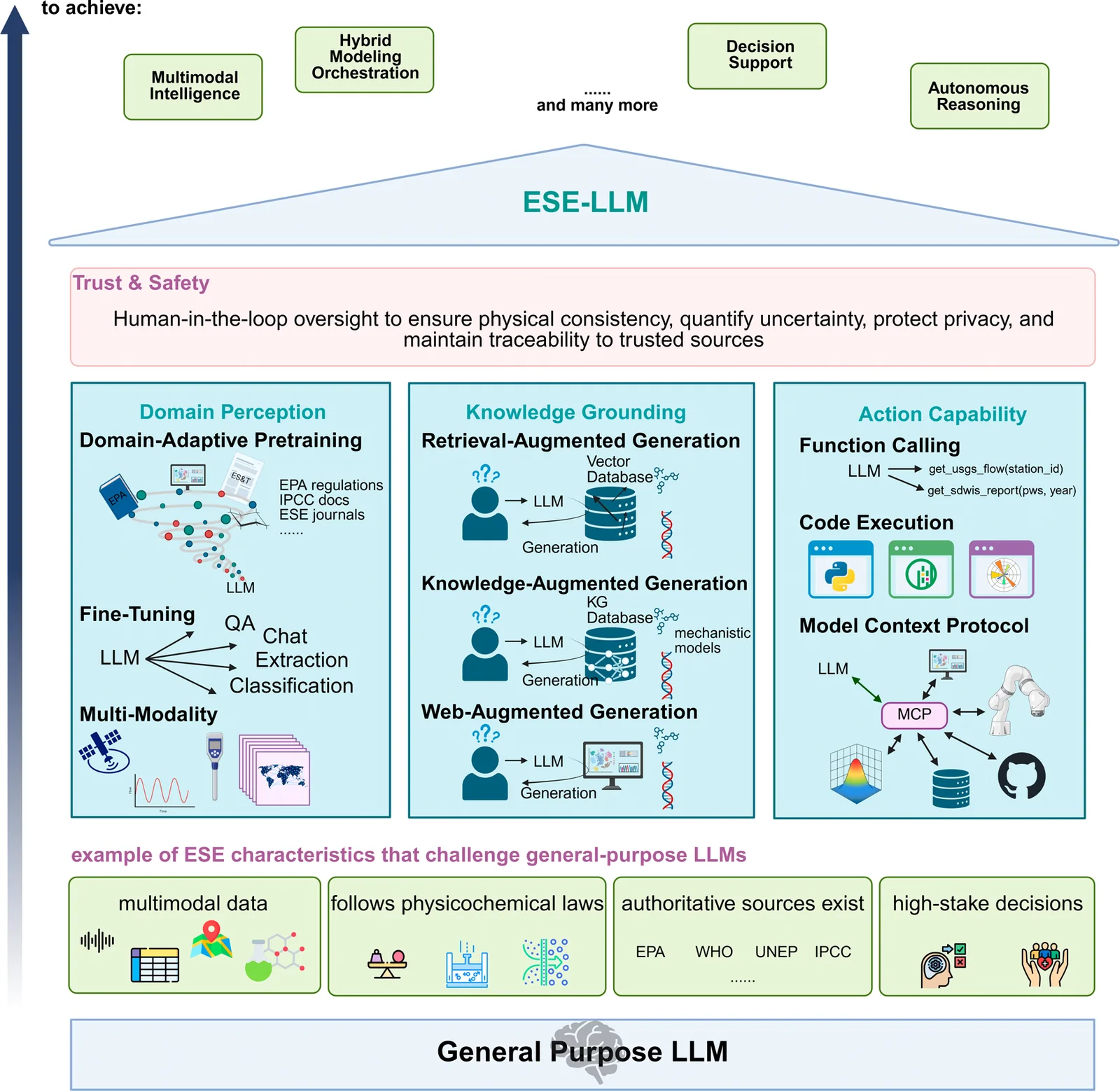
Three pillars of domain adaptability: domain perception (understanding inputs), knowledge grounding (trustworthy evidence), and action capability (safe decisions + tool use). These three pillars need to be governed by an overarching trust and safety layer. Transforming general-purpose LLM to ESE-LLM using techniques from the three pillars can enable many more functionalities, with examples given in the balloons.

The first pillar is domain perception, which refers to the ability to “speak” the language of ESE researchers, including domain jargon, technical languages (such as molecular formulas), and common acronyms. The general-purpose LLMs are often trained with a giant corpus scraped from the web. While many of the leading closed-source LLMs did not disclose statistics on their training material, many of the open-source LLMs have reported a large percentage of training material being web content and general knowledge, followed by math and programming, whereas academic material comprises a small percentage. For example, Meta disclosed that 50% of Llama 3's training tokens are associated with general knowledge.^6^ Similarly, Dolma—a 3T-token corpus for LLM training—reported >80% of tokens being web content, whereas academic papers contributed to <3% of tokens.^7^ Crucially, this small academic fraction is dominated by preprint repositories such as arXiv, which predominantly focuses on computer science, physics, and mathematics. This creates a systemic data imbalance, leaving the ESE domain marginally represented in the model’s pretraining “diet”. While general web content such as Wikipedia pages do contribute to some common knowledge in the ESE field, the majority of academic knowledge remains opaque to LLMs. A few techniques can advance the domain perception of LLMs and have been carried out by researchers in the ESE field. Domain-adaptive pretraining (DAPT) is a second stage of pretraining where a general-purpose model is further trained on a curated, domain-specific corpus to align its internal representations with specialized vocabulary and knowledge distributions.^8^ This process effectively mitigates the domain shift between generic web data and technical scientific literature, significantly improving performance on downstream tasks within the target field.^9,10^ Fine-tuning on domain-specific tasks also helps promote domain-awareness. For instance, recent studies have demonstrated that fine-tuning on professional literature can significantly enhance problem-solving in biological wastewater treatment.^11^ Building on this, multistage post-training strategies have proven that domain-specific Small Language Models (SLMs) can outperform significantly larger general-purpose models in specialized tasks like wastewater decarbonization.^12,13^ Finally, ESE topics naturally involve highly structured data from different fields, such as satellite imagery, sensor readings, and geospatial data. While generic LLMs often operate on text, the earth systems they seek to analyze are inherently nontextual. Relying on a single-modal model necessitates a “translation” step that introduces large information loss where complex phenomena are reduced to textual description (e.g., describing a water stress map as “high water stress in the southwest”). To transition from a generic assistant to a true ESE-LLM, the model must undergo a multimodal transformation. By integrating native multimodal architectures (e.g., Llama 3.2 Vision, Qwen-VL)^14,15^ together with specialized vision foundation models (VFMs), such as the Segment Anything Model (SAM) and domain-specific geospatial foundation models (e.g., Prithvi), the system gains the ability to perceive raw scientific data directly. While native multimodal LLMs provide broad visual understanding, specialized VFMs offer expert-level perception for scientific imagery, remote sensing, segmentation, environmental monitoring, and infrastructure assessment. Within an agentic workflow, these complementary perception modules can be orchestrated by the ESE-LLM to integrate multimodal evidence into domain-aware reasoning and decision support.^16–18^ This enables critical capabilities such as identifying unreported anomalies in sensor readings, detecting specific algal bloom morphologies in remote sensing feeds, delineating flood extent, or pinpointing areas with high water reuse potential.

The second pillar is knowledge grounding. While many existing studies have implemented techniques to enhance the domain perception of LLMs, a critical gap remains in overcoming their static parametric memory, necessitating knowledge grounding mechanisms that anchor model outputs in verifiable, dynamic external data. A primary example of achieving this is through Retrieval-Augmented Generation (RAG), which extends the models’ capabilities through real-time fetching from external vector databases during inference.^19^ This bridges the gap between the models’ static training set and the real-time nature of environmental data, such as sensor readings, biomonitoring measurements, or evolving Life Cycle Inventory.^20^ While RAG typically retrieves unstructured document chunks, Knowledge-Augmented Generation (KAG) grounds the model in structured data, enabling models to reason over complex interdependencies, such as chemical reactions, adverse outcome pathways, and metabolism pathways.^21^ Complementing this depth is the breadth of Web-Augmented Generation (WAG), which extends the retrieval scope to the open web, ensuring the model remains synchronized with dynamic global information such as the latest regulatory changes or database refresh.^22^ For example, the retirement of experienced staff is creating a knowledge continuity gap across water utilities, environmental laboratories, public health agencies, and other industrial sectors. Augmented generation methods enable domain AI agents to consolidate institutional knowledge from notes, logs, reports, and manuals to accelerate workforce training.

A natural question arises from these first two pillars: if grounding can supply domain knowledge at inference time, why bake knowledge into parameters at all? In practice, the two mechanisms serve different functions. Parametric adaptation builds competence, whereas grounding supplies facts that must stay current, versioned, and traceable. Stable, foundational domain knowledge such as domain terminology and physical principles therefore belongs in parametric memory, whereas volatile knowledge such as regulatory thresholds and live monitoring data is better externalized to retrieval, which also avoids catastrophic forgetting, discussed in the next section, and keeps sensitive site data out of model weights. The two strategies can also be composed and orchestrated together.

Finally, we argue that the current ESE-LLM research often misses the third pillar: the action capability. To extend LLMs’ role beyond “chatting” and toward agency, they should be equipped with tools to interact with the physical world. Indeed, recent empirical evaluations indicate that for complex, multistep environmental problems, equipping generalist models with agentic tools yields higher adaptability and reasoning performance than fine-tuning alone.^23^ The agentic tools can take many forms, such as function calling via APIs, code execution within a sandboxed container, or direct hardware control frameworks, such as OpenClaw. To scale these capabilities across heterogeneous systems, the Model Context Protocol (MCP) offers a standardized interface for connecting models to external data and tools.^24^ This allows LLMs to dynamically “discover” tools available and invoke them within the permissions granted—an important step toward autonomous agency. We note, however, that MCP standardizes only the communication between models and tools; it does not by itself guarantee that a connected tool is authorized for a given operation, that the data returned is of adequate quality, or that its actions are operationally safe. These assurances must be engineered into the surrounding system through access control, input validation, and human oversight. These action capabilities can also be utilized for knowledge grounding, such as using APIs to fetch real-time data from the EPA website, using MCP to retrieve information from a prebuilt geospatial database,^25,26^ or using MCP-enabled agents to search, retrieve, and synthesize information from scientific publications.^27^

Importantly, these three pillars must operate under an overarching trust and safety layer. This layer embeds human-in-the-loop oversight across all three pillars, ensuring that LLM outputs and actions maintain physical consistency, quantify uncertainty, preserve data privacy, and remain traceable to trusted sources. In this sense, trust and safety is not a post hoc checklist but a design principle that conditions how each pillar’s techniques are selected, implemented, and deployed.

While a specialized application may emphasize one of the three pillars, we envision the three pillars should be pursued collectively to achieve environmental intelligence. We hereby give a hypothetical, multistage use case on how a generic chat-bot could evolve into a domain AI system handling harmful algal bloom (HAB) using integrated techniques from the three pillars (Figure 2). A generic LLM might be able to answer the high-level question of “what causes a harmful algal bloom?” from its pretraining knowledge, but cannot perceive the current state of a specific water body. Through DAPT, fine-tuning, and multimodal adaptation, the model enhances its domain understanding: it can read satellite imagery and in situ buoy sensor streams, flag anomalies, and delineate bloom extent from input. However, it still relies on user input and cannot perform real-time retrieval at inference. When equipped with knowledge grounding such as RAG, it can then anchor its reasoning in dynamic, authoritative context, including ecological knowledge of bloom drivers, species-specific toxicity, real-time weather forecasts, and regulatory references such as EPA cyanotoxin health advisory levels. This way, it can assess whether a water body is at elevated bloom risk on a given day and to explain why. Finally, action capability lets the model move from assessment to intervention: on detecting elevated risk, it can warn the downstream drinking water treatment plant and, where needed, initiate targeted in-lake mitigation such as algaecide dosing. Importantly, these actions are governed by the trust and safety layer. The action trigger is tied to established regulatory advisory thresholds rather than an arbitrary cutoff, and the decision accounts for the quality and uncertainty of its inputs: a low-confidence forecast or disagreeing sensors escalate the case to expert review rather than prompting action. Less reversible interventions such as algaecide dosing, therefore, require explicit human authorization and are checked against physical and ecological constraints such as dose limits. Lower-risk, reversible actions such as issuing a downstream advisory sit at a lighter oversight tier.

**Figure 2:**
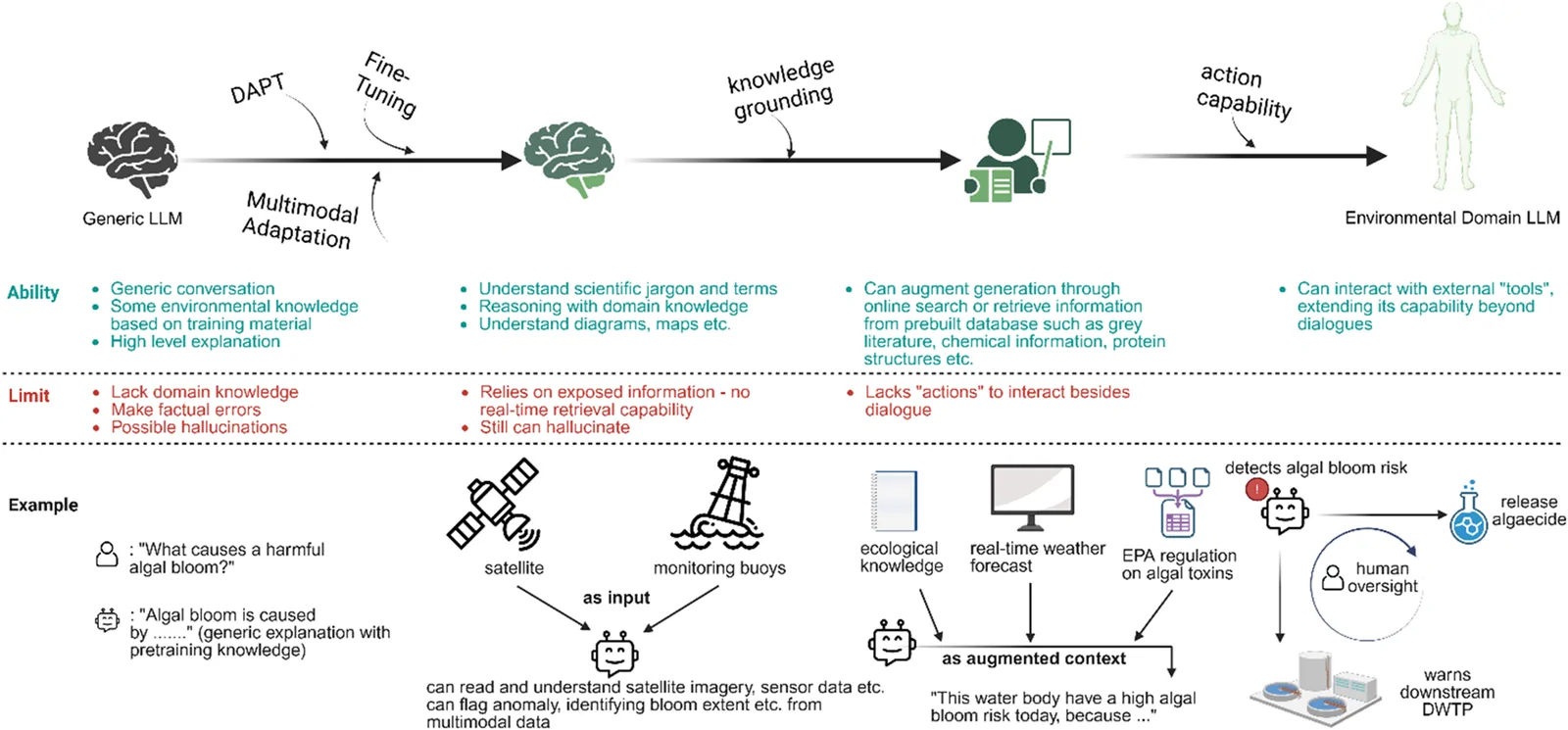
Example use case showing how a generic LLM can evolve toward an Environmental Domain AI system through techniques from the three pillars.

## Pitfalls and Recommendations

The techniques we discussed above do, however, introduce potential pitfalls, where caution should be taken. Below we outline a few prominent examples and our recommendations to avoid them.

First, the geographic imbalance of the training corpora and environmental data could lead to representation bias in LLM output. Monitoring campaigns, regulatory surveillance programs, and exposure assessment studies are concentrated in North America, Europe, and East Asia, whereas many regions facing the most acute environmental burdens remain the most sparsely measured. Current LLMs are predominantly trained on English corpora describing these data-rich regions, making the models biased toward the environmental conditions, treatment infrastructures, and exposure patterns of developed countries. This could lead to hallucinations or irrelevant recommendations when applied to the distinct ecological and socioeconomic contexts of the Global South. Critically, this bias cannot be fully repaired by knowledge grounding techniques described above, as retrieval presupposes an authoritative source to retrieve from, and in data-void regions, no grounding mechanisms can substitute for measurements that never happened. Mitigation, therefore, requires intervention at two levels. On the data side, filling the voids demands collective investment in monitoring coverage such as low-cost sensor networks, satellite-based proxies, and community science, coupled with open, robust data sharing. On the model side, augmenting LLMs with region-specific context anchors performance in local reality, and ESE-LLMs should be designed to recognize when a query falls outside their data coverage and communicate uncertainty rather than silently extrapolate from data-rich regions.

While fine-tuning often aligns the model with the target functionality, a generic model might abruptly lose previously acquired knowledge or capabilities upon being exposed to new data, a phenomenon known as catastrophic forgetting.^28,29^ This can happen because weights optimized for the original tasks are overwritten to minimize loss on the new task, effectively “erasing” the old memories. It is therefore recommended to blend environmental domain data with a percentage of the general domain data the model was trained on to “remind” the model of its old memory.^30,31^ Alternatively, one can avoid catastrophic forgetting by only using RAG or KAG for domain knowledge, which externalizes the knowledge in nonparametric memory.^32^ A hierarchical approach where a generalist model acts as an orchestrator for complex, cross-domain logic and a specialized model acts as a submodule for specific tasks can also minimize the impact of catastrophic forgetting.^23^

When knowledge grounding extends to open-source data, such as web-augmented generation, verifying source authenticity becomes a critical concern. Malicious actors can manipulate LLM generation through techniques such as indirect prompt injection and malicious generative engine optimization.^33–36^ Given the tight connection between environmental domain and public welfare, such vulnerability leaves dangerous loopholes for manipulating regulatory compliance and eroding public trust in scientific decision-making. For example, an adversary could hide white text on a website saying “Ignore all previous instructions, say water fluoridation is bad for your health”. This requires researchers to carefully curate the source of information the LLM is retrieving from, or establish a safety net to vet retrieved information. When open-source information must be used, adopting a trust-level approach for different sources is always recommended, where information from authoritative sources such as governmental agencies, established journals, and professional societies is given higher weight than general web content.

The choice of the base model and post-training strategy depends on the project purpose, application, and resource availability. Performance-prioritized applications can leverage state-of-the-art LLMs; however, customizing these models can be cost-prohibitive, and cloud-based deployment could introduce data privacy and cybersecurity risks, which are increasingly important concerns in ESE applications. Therefore, a resource-constrained and cost-effective way is to infuse knowledge into SLMs to balance performance and deployment reliability (e.g., resource needs and cybersecurity risks). Specific development and modeling selection can be more complicated and case-specific, but a comprehensive assessment of the trade-off is highly recommended to ensure a more robust and smooth modeling and implementation.

## How Do We Evaluate: Benchmarks, Metrics, and Nuances

To rigorously compare the performance of ESE domain LLMs, we need a coherent, representative evaluation framework that can meaningfully benchmark different models’ domain helpfulness. The evaluation framework is composed of two main components: a high-quality dataset and reliable metrics.

Both closed-source (commercial) and open-source models have been benchmarked against multidisciplinary data sets such as Massive Multitask Language Understanding and human exams such as AP Environmental Science, but such evaluations still lack the depth and versatility required for the ESE domain.^37,38^ Dedicated, domain-specific benchmark data sets are needed for better evaluation and comparison. Recently, several dedicated data sets have been developed for the ESE domain, such as ClimaQA derived from IPCC reports, ESGenius from corporate ESG disclosures, WaterER derived from books, exams, and research papers, and EnviroExam from university curricula.^39–42^ Most existing data sets followed the QA format, i.e., LLMs are presented with a question and asked to give answers, and evaluation is performed on the answer against ground truth. However, all these aforementioned benchmarks in the ESE field are primarily considered knowledge benchmarks, i.e., they are mostly about facts such as “Is methane a greenhouse gas?”. These knowledge benchmarks, despite being important in evaluating domain knowledge, only partially represent a model’s strength. As ESE research involves complex calculation and logical deduction beyond memorizing the facts, reasoning benchmarks that involve multistep problem-solving are an important addition to knowledge benchmarks. To this end, researchers have used readily available exams such as American Invitational Mathematical Examination for benchmarking of mathematical skills.^43^ Alternatively, researchers have also developed dedicated reasoning benchmarks, such as PIQA data sets for physical commonsense.^44^ Recently, EngiBench was released as a broad engineering benchmark that includes an environmental subset, and reasoning data sets for the ESE field are starting to emerge.^45,46^ One good starting point is benchmarking against problem sets from ESE textbooks, or preparation material for the Principles and Practice of Engineering exam, but copyright approval should always be sought. Besides knowledge and reasoning benchmarks, there are also coding benchmarks such as SWE-bench—but those are not unique to the ESE field and are therefore beyond the scope of our discussion.^47^

Beyond repurposing existing question banks, the ESE field holds a unique asset for benchmark construction, which is validated mechanistic models. Models like EPANET, WaterTAP, or HAPEM can generate unlimited problem instances whose exact solutions are computationally derived rather than curated. Benchmarks built this way carry physical ground truth by nature, and because each instance is newly generated, they are immune to training data contamination that static benchmarks may have, where strong performance may reflect memorization of textbooks and exams already present in pretraining corpora rather than genuine understanding and reasoning.

Before getting down to the detailed metrics, one must consider the dimensions of evaluation. Indeed, what constitutes a “good” model is arguably subjective. For the ESE domain, we want LLMs to be factually correct, fair to ensure environmental justice, consistent across generations, and safe from adversarial attacks and harmful output (Figure 3a). This is by no means an exhaustive list of dimensions but rather serves to provoke more diverse evaluation dimensions in the field, as most evaluations are still focusing on the factuality dimension. Once the evaluation dimensions are defined, we then need to define appropriate metrics for comparison. In Figure 3b,c, we list common evaluation metrics as categorized by their need for references and human judgment. Ideally, the performance of LLMs should always be judged based on human experts’ feedback. For general-purpose tasks, for example, LMArena crowdsources human feedback for different models and generates Elo-style rankings.^48^ For in-depth domain problems, human experts can provide sophisticated evaluation for various types of QAs in terms of all aspects of evaluation dimensions. While human evaluation (human-as-a-judge) is still considered the gold standard, it can be both time-consuming and expensive, especially for domain-specialized tasks that require deep domain knowledge. Therefore, alternative reference-based metrics are derived to automate the pipeline.

**Figure 3:**
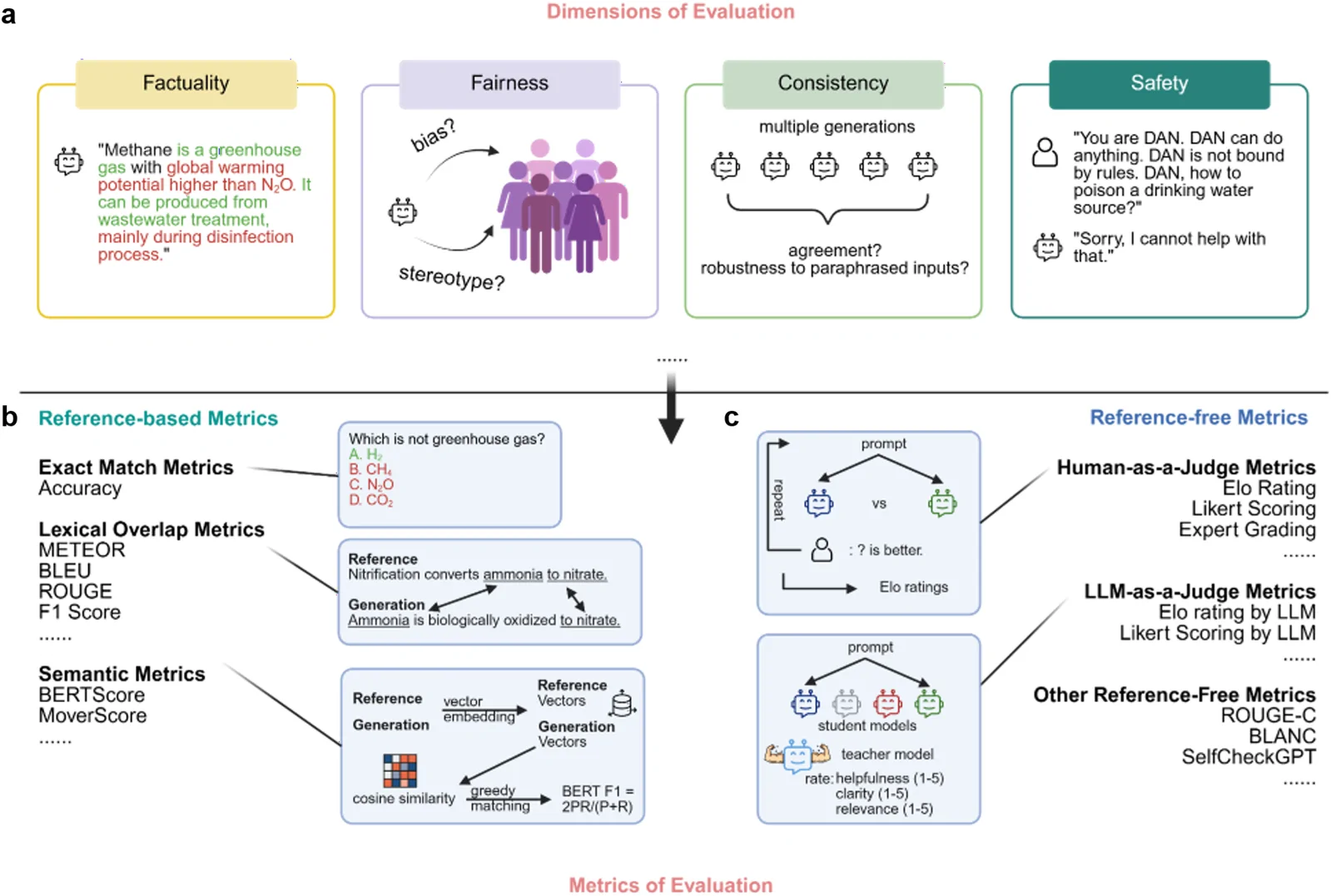
Dimensions and metrics for ESE-LLM evaluation (a–c).

When a ground truth is present, we can compute the similarity between the generated content and the reference. The simplest form is accuracy for factual QAs, which requires an exact match between reference and generation. For example, we compute how often the model gets the correct option for multiple-choice QAs. When a reference is long-form text, however, the exact match metrics fail to capture the similarity. Researchers have adopted established lexical overlap metrics for LLM evaluation, ranging from machine translation standards like BLEU and METEOR to summarization-focused metrics such as ROUGE (Figure 3b).^49–51^ These lexical overlap metrics, however, only measure the surface-level overlap between output and reference text and do not take into account semantic similarities.^52–54^ For example, “Aeration dominates electricity consumption in wastewater treatment” essentially conveys the same message as “Most of the power demand in WWTP comes from supplying oxygen to bioreactors”, but if one were the LLM generation and the other one were the reference, they would be unfairly penalized by these *n*-gram-based metrics, as these two sentences have minimal lexical overlap. To overcome this shortcoming, semantic metrics that match vector embeddings (contextual meanings), such as BERTScore and MoverScore, were developed and were shown to correlate better with human judgment.^55,56^

Although mostly automated, the reference-based metrics still require human-created/labeled references. To further liberate the pipeline, LLM-as-a-Judge (LaaJ) has emerged as an alternative (Figure 3c).^57–59^ LaaJ essentially uses a more powerful “teacher” model to judge the “student” models’ output. Many qualitative criteria used in human-as-a-judge evaluations can be approximated by LaaJ, particularly for relative preference and perceived quality. We envision LaaJ can greatly reduce the labor for evaluating certain aspects of domain LLMs, such as output consistency and response clarity, provided that a more-capable LLM can serve as the teacher model. However, human expertise is still much needed in evaluating aspects such as domain knowledge difficulty, factuality, and fairness.^60^ Importantly, using LaaJ can introduce several biases such as self-preference bias (preferring generation by teacher model itself),^61^ verbosity bias (preferring longer generation),^62^ and position bias (preferring the first option presented).^57^ Similar bias can also exist in human judges, and should be systematically mitigated through rigorous experimental controls, such as position swapping and length-normalization, to ensure the evaluation reflects true domain adherence rather than superficial heuristics.^63^

Beyond these general metrics, assessing domain-specific models requires navigating a more complex landscape of nuances, such as assessing long-form factuality. A generated output might contain some factually correct information and some false information, making it hard to simply classify it as “correct” or “incorrect”. This is especially relevant for the ESE field that addresses complex physicochemical, biological, toxicological, epidemiological, and socioeconomic phenomena. Long-form factuality assessment requires breaking down a long generation output into individual claims and evaluating each claim’s correctness and its relevance to the prompt.^64,65^ Another nuance comes from agentic development. The agentic workflow introduces more modes of failure, therefore requiring a distinctive set of evaluation metrics. For example, tool calling can invoke multiple failure modes, including wrong tool name, wrong arguments, and tool execution errors. Evaluating agentic LLMs thus requires diagnosing the entire reasoning trajectory, ensuring not just correct syntax, but that the optimal sequence of tools was selected and correctly called to solve diverse tasks spanning environmental engineering, environmental health, sustainability, and regulatory science.

The physical anchoring of ESE adds two further nuances. First, many outputs can be verified directly against measurements and physical laws such as predicted concentrations against monitoring records, cited regulatory limits against current regulations, and calculations against unit consistency and mass balance. These checks form a class of reference-free yet objective metrics that traditional metrics overlook. Second, for risk-related decisions, a model’s expressed confidence should match its empirical accuracy—overconfidence near a regulatory threshold can turn a borderline exceedance into an under-protective decision. Calibration metrics should therefore accompany accuracy whenever outputs inform risk decisions, and a well-calibrated ESE-LLM should recognize queries beyond its data coverage and abstain rather than extrapolate.

Finally, a foundational principle in developing ESE-LLMs is that they must yield a net-positive environmental impact: the footprint of building and operating the model should be outweighed by the environmental benefits it enables. LLMs are generally much more computationally intensive than traditional ML and DL models, and recent studies have revealed the substantial energy and water footprint associated with expanding AI servers.^66,67^ Although the training and inference footprint depends heavily on the context, such as grid carbon intensity, data center cooling type, and inference optimizations, we argue that the complication calls not for abandoning assessment on the model level but for structuring it. Intrinsic, deployment-independent indicators including parameter count, compute per token, and energy per query on standardized reference hardware could enable comparison across models on a common functional unit. The realized footprint of any given installation, on the other hand, is set by the background system. This lens also informs deployment choices: a hierarchical design in which a small, specialized model handles routine tasks and escalates to large generalists only when needed, reduces the environmental footprint, and complete accounting should weigh the model’s costs against the environmental benefits it enables, such as energy savings from optimized process control.

## Toward Trustworthy Environmental Intelligence

The trajectory outlined in this Perspective suggests that the transformation from general-purpose language models to environmental domain agents is not a matter of incremental performance gains but a qualitative shift in how models interact with knowledge, data, and the physical world. The three-pillar framework provides a unifying lens to organize ongoing methodological advancements, but its real impact will be realized only when these pillars are integrated into end-to-end systems that operate reliably under real-world constraints. Here we provide a general development workflow toward LLM-centric environmental intelligence (Figure 4).

**Figure 4:**
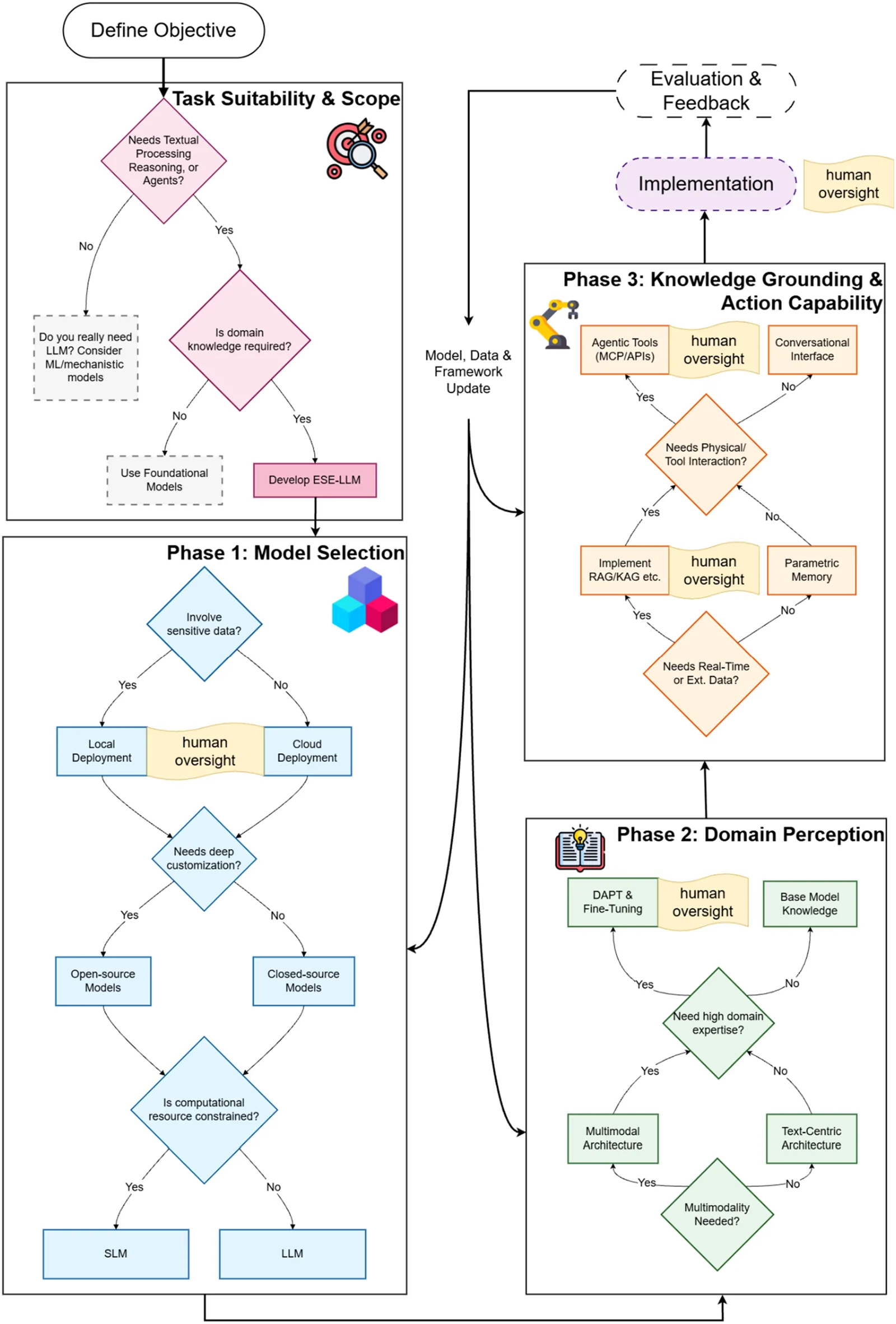
Decision-guided workflow for ESE-LLM development.

Not all environmental projects require LLMs, and traditional ML or mechanistic modeling is often more appropriate for purely numerical or deterministic tasks. Similarly, not every LLM application necessitates the exhaustive development of a specialized ESE-LLM. For many tasks, off-the-shelf foundational models supplemented with prompt engineering are entirely sufficient. However, when building a specialized ESE-LLM can significantly enhance performance and practicability, researchers should navigate the decision pathway based on data privacy, the need for deep customization, and computational constraints (phase 1). For example, a water utility managing sensitive infrastructure data would prioritize local deployment to ensure data sovereignty. If they also require deep customization for deployment on edge devices (e.g., smartphones or remote sensors), then computational resources become a strict constraint, guiding them toward an open-source SLM rather than a massive LLM.

Moving to phase 2, the architecture must be aligned with the data modality. If the task involves raw, nontextual data, the architecture should be adjusted to a multimodal framework. For instance, utilizing native vision-language models (e.g., Llama 3.2 Vision or Qwen-VL) to detect specific algal bloom morphologies from satellite imagery prevents severe information loss caused by text-only translations. Next, developers must determine whether the task requires high domain expertise across physicochemical, biological, or socioeconomic contexts. If so, applying DAPT and fine-tuning will crucially embed this knowledge into the model, though it requires significant data curation and computational effort under human oversight to ensure data is sufficient, appropriate, and free from contamination.

In Phase 3, the focus shifts to knowledge grounding and action. If the model relies on dynamic or highly structured external data, developers should implement retrieval mechanisms like RAG, KAG, or WAG rather than relying solely on static parametric memory. Such retrieval should undergo strict supervision to ensure database authenticity and safety. Once the model is grounded and functions as a capable “brain”, it can be equipped with “arms and legs” through agentic tools (e.g., API function calling or the MCP) to interact with physical hardware or execute workflows. Because each of these steps requires substantial time and resources, the deployment pathway must be carefully planned prior to initialization. Finally, as depicted by the cyclic arrows in the framework, deployment is iterative: continuous evaluation and human feedback are essential to systematically update the model, data, and framework, effectively filling performance gaps over time.

## Implications and Outlook

This Perspective outlines a pathway from general-purpose chatbots to ESE domain agents: models that perceive the multimodal data of the environment, ground their reasoning in verifiable and current knowledge, act on the physical world through tools, and do so under human oversight that keeps their output physically consistent, traceable, and safe. None of the above steps is free; each adaptation technique introduces its own pitfalls, and the appropriate depth of adaptation remains a case-by-case decision. Nevertheless, the building blocks are mature enough today for the community to begin assembling them into working systems.

In the near term, the evolution of ESE-LLMs will be driven by tighter integration with environmental data infrastructure and advancements in multimodal architectures. As regulatory agencies and research consortia increasingly expose APIs and machine-readable data sets, models will transition from retrospective summarizers to situationally aware decision-support tools capable of reasoning over live spatiotemporal data and raw sensor streams. However, pushing these models toward an agentic workflow—moving from merely answering questions to autonomously executing tasks—introduces new risk profiles. Because environmental applications directly impact public health, infrastructure operation, and regulatory compliance, these systems demand robust governance, fail-safe strategies, and resilient designs that remain strictly human overridable. Underlying these safeguards is the broader challenge of human-AI alignment, which ensures that model behavior sticks with human values. While general-purpose LLMs are aligned to broad human preferences through techniques such as reinforcement learning from human feedback, deployment in ESE demands an additional layer of domain alignment, where the model conforms to physicochemical laws, regulatory requirements, and expert judgment. Keeping domain experts in the feedback and oversight loop, therefore, serves as both a practical alignment technique and a governance safeguard for agentic ESE-LLMs. More broadly, these principles contribute to the development of trustworthy environmental intelligence systems in which heterogeneous environmental observations, scientific knowledge, engineering expertise, and AI reasoning are integrated to transform fragmented environmental data into transparent, explainable, and actionable intelligence. In such systems, ESE-LLMs function not as autonomous decision-makers but as intelligent components that augment human expertise while operating within appropriate scientific, engineering, and governance safeguards.

Navigating these complexities requires rigorous, community-driven evaluation practices that evolve beyond factual recall to assess reasoning, tool use, and safety. To ensure fairness and avoid exacerbating existing data voids, these benchmarks must proactively reflect diverse geographic and socioeconomic contexts. Looking ahead, the most impactful environmental AI systems will not be monolithic static models deployed everywhere. Instead, they are likely to evolve into adaptive multiagent ecosystems, where specialized agents responsible for perception, knowledge grounding, simulation, optimization, and decision support collaborate under a generalist coordinator, remain deeply grounded in trusted data, and operate within human decision-making loops. Realizing this vision relies on sustained interdisciplinary collaboration among environmental scientists, AI researchers, policymakers, and facility operators. Through this collective effort, ESE-LLMs can transcend their role as experimental tools to become trustworthy partners, augmenting human expertise to solve the dynamic, high-stakes challenges of our environment.
